# Optimization strategies for ultrasound zoning of cervical lymph nodes in esophageal cancer: a precision medicine approach

**DOI:** 10.3389/fonc.2025.1596770

**Published:** 2025-08-06

**Authors:** Yi Zhu, Yuting Fan, Xinyi Luo, Hainan Chen, Shuoming Liang, Jicheng Xiong, Hiroyuki Daiko, Xuefeng Leng

**Affiliations:** ^1^ Department of Ultrasound, Sichuan Clinical Research Center for Cancer, Sichuan Cancer Hospital & Institute, Sichuan Cancer Center, Affiliated Cancer Hospital of University of Electronic Science and Technology of China (UESTC), Chengdu, China; ^2^ Department of Thoracic Surgery, Sichuan Clinical Research Center for Cancer, Sichuan Cancer Hospital & Institute, Sichuan Cancer Center, University of Electronic Science and Technology of China (UESTC), Chengdu, China; ^3^ Department of Esophageal Surgery, National Cancer Center Hospital, Tokyo, Japan

**Keywords:** esophageal cancer, cervical lymph nodes, supraclavicular lymph nodes, ultrasound diagnosis, zoning, staging, treatment

## Abstract

Esophageal cancer is a common malignancy with high incidence and mortality rates. Its pathological types vary by region, with squamous cell carcinoma predominant in Asia and adenocarcinoma in Western countries. Accurate staging before treatment is crucial for selecting appropriate therapeutic strategies. The existing international staging systems primarily include the American Joint Committee on Cancer and Union for International Cancer Control (AJCC/UICC) system and the Japanese Esophageal Society (JES) system. However, these systems differ in lymph node definitions and zoning, particularly regarding the classification and management of supraclavicular lymph nodes, which remains a contentious issue. Accurate zoning of the supraclavicular and cervical lymph nodes directly impacts treatment decisions for esophageal cancer, making precise ultrasound-based zoning diagnosis essential. Currently, the cervical lymph node zoning for esophageal cancer often follows the ultrasound standards established by the American Head and Neck Society and the American Academy of Otolaryngology-Head and Neck Surgery (ASHNS/AAO-HNS). However, the applicability of these standards to esophageal cancer is still under debate. Notably, the challenges associated with cervical lymph node zoning in esophageal cancer are particularly prominent in East Asia, especially in China and Japan, where squamous cell carcinoma is the predominant histological type and the JES staging system is widely adopted. In this system, supraclavicular lymph nodes are considered surgically resectable regional nodes, and their dissection is recommended as it may offer survival benefits. In contrast, clinical practice in Western countries primarily follows the AJCC/UICC staging system, which classifies supraclavicular lymph nodes as distant metastases (M1), generally precluding surgical intervention. These geographical and conceptual discrepancies in staging and treatment strategies highlight the urgent need to establish a globally applicable and standardized ultrasound-based lymph node zoning approach. This article aims to explore the optimization of ultrasound zoning for cervical lymph nodes in esophageal cancer based on literature and clinical practice, providing insights for precise staging and optimal treatment.

## 1 Distribution characteristics, zoning definitions, and clinical significance of cervical lymph nodes in esophageal cancer

### 1.1 Distribution characteristics and anatomical basis of lymph node zoning in esophageal cancer

The lymphatic metastasis of esophageal cancer exhibits significant site dependency and longitudinal distribution patterns, characterized by regionality, bidirectional spread, and skip metastasis (1, 2). The longitudinal lymphatic drainage of esophageal cancer originates from the lamina propria of the esophageal mucosa. Cancer cells penetrate the muscularis mucosae and enter the submucosal and deeper layers, spreading upward and downward along the lymphatic network (3, 4). This leads to metastatic pathways extending to the cervical region for upper esophageal tumors, while lower esophageal tumors may spread to the abdominal region. This characteristic longitudinal dissemination may result in distant regional lymph node metastases without involving distant organs in the traditional sense. Cervical lymph node metastases in esophageal cancer are primarily concentrated in the tracheoesophageal groove, the supraclavicular margin, the inferior margin of the cricoid cartilage, and the trapezoidal area medial to the accessory nerve. These include the cervical paraesophageal lymph nodes (bilateral No. 101 nodes) and the bilateral supraclavicular lymph nodes (bilateral No. 104 nodes). The cervical paraesophageal lymph nodes are distributed above the thoracic inlet of the esophagus, medial to the bilateral common carotid arteries, within the tracheoesophageal groove, and below the inferior margin of the cricoid cartilage in the cervical paraesophageal region. A portion of the lymphatic fluid around the esophagus drains through the cervical paraesophageal lymph nodes into the lymphatic ducts and subsequently into the venous angle, increasing the likelihood of metastasis in this region.

The supraclavicular lymph nodes are located posterior to the clavicle and below the cricoid cartilage, along the transverse cervical vessels. Their efferent ducts converge into the subclavian trunk, which ultimately drains directly into the thoracic duct or the right lymphatic duct at the venous angle. Due to their close anatomical relationship with the thoracic duct and other lymphatic trunks draining into the venous angle, the supraclavicular lymph nodes are prone to lymphatic metastases from esophageal cancer. The supraclavicular lymph nodes belong to the cervical lymphatic region and serve as critical metastatic sites for upper and middle esophageal tumors. In the staging and treatment decision-making process for esophageal cancer, metastases to the supraclavicular lymph nodes hold significant importance, with varying definitions in different staging systems (5). Research has demonstrated that the pattern of cervical lymph node metastasis in patients with cervical esophageal squamous cell carcinoma (ESCC) significantly impacts overall survival (OS). The 5-year OS rate for patients with No. 101 (cervical paraesophageal lymph node) metastases is 21%, while it is 34% for those with No. 104 (supraclavicular lymph node) metastases. This finding highlights that the prognosis for No. 101 metastases is significantly worse than for No. 104 metastases, underscoring the need for more precise and individualized early diagnosis and management of cervical paraesophageal lymph nodes (6).

A deeper understanding of the distribution characteristics and metastatic pathways of lymph nodes in esophageal cancer can aid in optimizing ultrasound zoning and diagnostic strategies for cervical lymph nodes, ultimately supporting individualized treatment decisions.

### 1.2 Three-field cervical lymphadenectomy and metastasis rates in esophageal cancer

In the early 1980s, Japanese studies revealed that 30%-40% of patients with thoracic ESCC exhibited cervical lymph node metastasis after surgery (7, 8). Led by Akiyama, Japanese surgeons first proposed the three-field lymphadenectomy (cervical, thoracic, and abdominal lymphadenectomy) for thoracic ESCC, expanding the surgical field from the thoracic and abdominal regions to include bilateral cervical lymph nodes. This systematic clearance of lymph nodes in the esophageal drainage regions aimed to reduce postoperative recurrence rates and improve survival outcomes (9). Cervical lymphadenectomy not only clarifies the metastatic patterns of thoracic ESCC but also enables the removal of clinically undetectable occult metastatic lymph nodes and micrometastases. This contributes to reducing local recurrence rates and improving long-term survival (10, 11). In 1991, studies conducted by the Japan Esophageal Society and the National Cancer Center of Japan demonstrated that three-field lymphadenectomy significantly increased lymph node metastasis detection rates compared to two-field lymphadenectomy (72.9% vs. 58.7%) (12). The expanded clearance also resulted in an upward shift in postoperative pathological staging. Among patients undergoing three-field lymphadenectomy, the supraclavicular lymph node positivity rate was 26%-27.4% (13, 14), and it was shown that three-field lymphadenectomy provided higher overall survival rates than two-field lymphadenectomy (13). Subsequent clinical studies further confirmed the survival benefits of three-field lymphadenectomy (12–15). In 1994, the Japan Esophageal Society (JES) established three-field lymphadenectomy as the standard surgical approach for thoracic ESCC.

An analysis of postoperative pathological data from patients undergoing McKeown esophagectomy at Fujian Cancer Hospital between 1999 and 2007 showed that the cervical lymph node metastasis rate for upper thoracic ESCC was 46.3%, with metastasis rates of 43.3% for bilateral No. 101 lymph nodes and 13.8% for bilateral No. 104 lymph nodes. The cervical lymph node metastasis rate for middle thoracic ESCC was 35.8%, with metastasis rates of 32.2% for bilateral No. 101 lymph nodes and 9.8% for the right No. 104 lymph nodes. The cervical lymph node metastasis rate for lower thoracic ESCC was 28.8%, with metastasis rates of 24.5% for bilateral No. 101 lymph nodes and 9.4% for bilateral No. 104 lymph nodes (16). Results from international cohort studies further illustrate that for upper thoracic ESCC patients, the cervical paraesophageal lymph node metastasis rate was 55% (34% on the right, 22% on the left), and the supraclavicular lymph node metastasis rate was 14% (9% on the right, 4% on the left). For middle thoracic ESCC patients, the cervical paraesophageal lymph node metastasis rate was 35% (24% on the right, 11% on the left), and the supraclavicular lymph node metastasis rate was 15% (10% on the right, 5% on the left). For lower thoracic ESCC patients, the cervical paraesophageal lymph node metastasis rate was 14% (10% on the right, 4% on the left), and the supraclavicular lymph node metastasis rate was 16% (13% on the right, 3% on the left) (3).

### 1.3 Zoning definitions and clinical significance of cervical lymph nodes in esophageal cancer staging systems

The Union for International Cancer Control (UICC) established the TNM staging system for tumors in 1968, which was integrated with the American Joint Committee on Cancer (AJCC) staging system in 1988 (17). In the 7th edition (2009) and the updated 8th edition (2017) of the AJCC/UICC TNM staging system, the N stage was reclassified based on the number of lymph node metastases (18, 19). Additionally, supraclavicular lymph node metastasis was grouped together with distant organ metastasis as M1 stage, classifying esophageal cancer with supraclavicular lymph node involvement as stage IVB, for which surgery is no longer recommended. It is noteworthy that the 8th edition TNM staging system was based on data from 22,654 esophageal cancer cases collected from 33 medical centers across 12 countries, with 70% of the data derived from Western countries and only 20% from Asia (20). However, the staging system lacks data supporting three-field lymphadenectomy, which introduces limitations in the definitions of regional lymph nodes and the rationale for N staging in esophageal cancer.

The Japanese Esophageal Society (JES) was established in 1969. In the 12th edition of the JES esophageal cancer staging system, released in 2022, JES aligned with the UICC and AJCC staging systems by adopting the number of lymph node metastases as the criterion for N staging, discarding the previous tumor location-based lymph node grouping standard (21, 22). In the updated staging system, supraclavicular lymph nodes are no longer considered regional lymph nodes for thoracic esophageal cancer; their metastases are classified as M1a, distinct from distant organ metastases (M1b). From a clinical treatment perspective, supraclavicular lymph node metastases are grouped with N2-N3 stages of esophageal cancer in the TNM staging system, emphasizing the potential value of their surgical clearance (21–24). The JES staging system aligns more closely with the characteristics of esophageal cancer in Asian patients, particularly those with squamous cell carcinoma. Given that esophageal squamous cell carcinoma in Asian populations tends to spread longitudinally to cervical and supraclavicular lymph nodes, the JES staging system considers these regions as local lymph nodes and encourages three-field lymphadenectomy. This approach contrasts significantly with Western staging systems. Moreover, the JES system is based on extensive clinical data from Asian patients, providing a more precise definition of regional metastasis and offering patients with cervical lymph node metastases the opportunity for surgical intervention. In comparison, Western staging systems tend to classify supraclavicular lymph node metastases as distant metastases, making them less applicable in Asian populations. Despite differences in the treatment principles and management of supraclavicular lymph node metastases between Western and Japanese guidelines, the consensus in Asia, particularly in China and Japan, is that supraclavicular lymph node metastases represent resectable regional lymph nodes with potential benefits from surgery. This viewpoint has also garnered support from some American scholars (25–28). Thus, accurate diagnosis of cervical lymph node metastases, including supraclavicular lymph nodes, is of paramount importance. It not only helps refine staging systems but also directly informs treatment decisions, enabling the formulation of more appropriate therapeutic strategies for patients.

To clarify the key differences between staging systems and their implications for ultrasound practice, a comparative summary is presented in **Table 1**.

**Table 1:** Comparative summary of JES and AJCC/UICC cervical lymph node classification and clinical implications.

Aspect	JES Staging System (East Asia)	AJCC/UICC Staging System (Western countries)	Clinical Implication	Ultrasound Zoning Challenge
Supraclavicular lymph nodes	Considered regional (No. 104), resectable	Considered distant metastasis (M1)	Impacts operability and curative intent	Current ultrasound zoning standards do not reflect JES No.104
Cervical lymph nodes (No. 101–104)	Detailed anatomical subdivision (e.g., bilateral compartments)	Not clearly specified; grouped under distant nodes	Affects surgical field and extent of lymphadenectomy	Lack of ultrasound equivalents for JES-defined zones
Imaging standards applied	JES-based anatomical mapping	AAO-HNS/ACR-based head and neck zoning	Geographic inconsistency in ultrasound interpretation	Difficult to standardize for global staging practice

## 2 Application of ultrasound in evaluating cervical lymph nodes in esophageal cancer

Similar to other solid tumors, treatment recommendations for esophageal cancer rely on accurate diagnosis and staging. The evaluation of cervical lymph node metastasis is critical for selecting appropriate treatment strategies. As a non-invasive and convenient imaging tool, ultrasound is widely used for detecting and diagnosing cervical lymph node metastases in esophageal cancer due to its high-resolution imaging capability for superficial lymph nodes. It provides effective support for precise staging and treatment decision-making.

Early and accurate detection of esophageal cancer plays a pivotal role in improving patient outcomes. Recent advances in precision imaging technologies have demonstrated great potential in enhancing diagnostic sensitivity and specificity for early esophageal lesions. Recent imaging innovations, including AI-assisted multimodal systems, spectrum-aided visual enhancement, and hyperspectral imaging, have improved early diagnostic accuracy (29–31). These advances highlight the need to refine ultrasound-based lymph node evaluation to support precise staging and treatment planning.

### 2.1 Fundamental ultrasound assessment criteria and diagnostic standards

**Figure 1:**
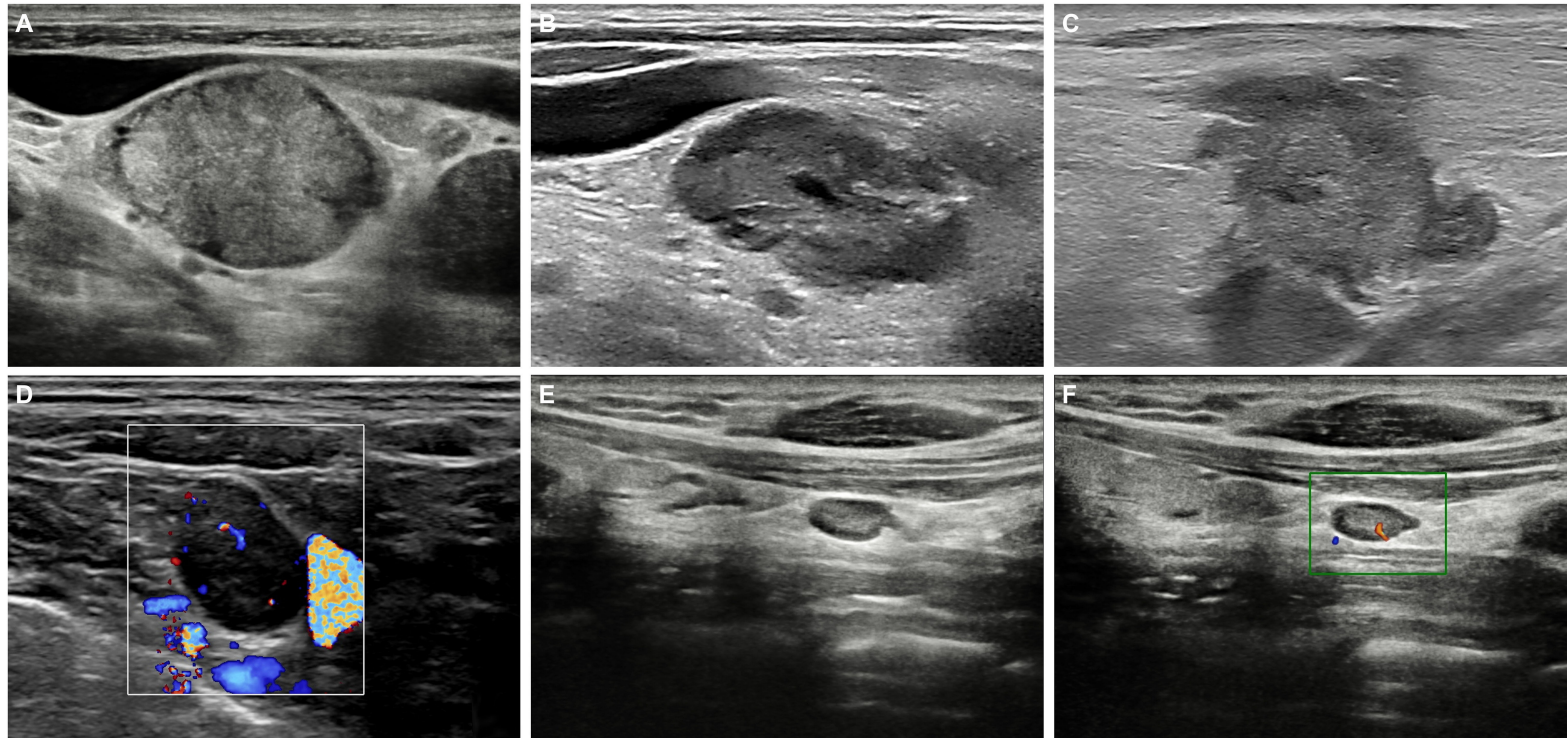
Ultrasound features of cervical metastatic and normal lymph nodes in esophageal cancer. **(A)** Metastatic lymph nodes have a short-axis diameter > 5 mm, appear hypoechoic, and are round in shape (long-to-short axis ratio ≤1), lacking a visible hilum structure. **(B)** Metastatic lymph nodes may contain areas of liquefaction or necrosis. **(C)** The borders of metastatic lymph nodes are often irregular. **(D)** Color Doppler Flow Imaging CDFI of metastatic lymph nodes shows abundant blood flow signals. Normal or reactive lymph nodes on B-mode **(E)** and CDFI **(F)**.

In the ultrasound evaluation of metastatic cervical lymph nodes in esophageal cancer, the following characteristics are commonly observed (**Figure 1**). 1) Size: Metastatic lymph nodes are typically enlarged, with a short axis usually exceeding 5 mm. Lymph node enlargement is one of the basic indicators for assessing malignancy. 2) Shape: Metastatic lymph nodes are often round or nearly round, with an aspect ratio (long-to-short axis ratio) close to or less than 1. In contrast, normal or benign lymph nodes are typically oval-shaped, with an aspect ratio greater than 1 (i.e., the long axis is significantly larger than the short axis). 3) Echogenicity: Metastatic lymph nodes usually appear hypoechoic or anechoic, indicating the potential presence of liquefaction or necrotic areas. Benign lymph nodes, on the other hand, are generally isoechoic and have a homogeneous appearance. 4) Margins: The margins of metastatic lymph nodes are often irregular, suggesting malignant infiltration. 5) Internal Structure: Metastatic lymph nodes often lack a hilum structure, which is characterized by the absence of the typical hyperechoic central area on ultrasound. 6) Vascular Pattern: In metastatic lymph nodes, tumor cell infiltration and neovascularization result in evenly distributed blood flow throughout the lymph node. This leads to the absence of the typical hilar blood flow pattern, presenting as abundant central blood flow signals. In contrast, normal or reactive lymph nodes typically show a hilar blood flow pattern on Color Doppler Flow Imaging (CDFI), with blood flow concentrated in the hilar region, forming strip-like or fan-shaped distributions. These differences in ultrasound characteristics can help distinguish between benign and malignant lymph nodes, thereby improving diagnostic accuracy.

### 2.2 Diagnostic performance of ultrasound in evaluating cervical lymph node metastasis in esophageal cancer

Ultrasound has high resolution for assessing the morphological characteristics, internal structure, and blood flow features of lymph nodes. Studies have reported that ultrasound demonstrates a sensitivity, specificity, and accuracy of 74.5%, 94.1%, and 87.6%, respectively, in detecting cervical lymph node metastases in esophageal cancer (32). A previous meta-analysis conducted by the authors indicated that ultrasound exhibits high diagnostic performance in evaluating cervical lymph node metastasis in esophageal cancer. When using a 5 mm threshold, sensitivity and specificity were 84% and 93%, respectively. When the threshold was increased to greater than 5 mm, sensitivity and specificity improved to 94% and 98%, respectively, suggesting that adopting a higher threshold could further enhance diagnostic accuracy (33).

Although CT and PET-CT are widely utilized, their performance in primary tumor staging is relatively poor, with accuracies below 50% (34). CT and PET-CT can complement endoscopic ultrasound in detecting lymph node metastases, with overall sensitivities of 50% and 57% and specificities of 83% and 85%, respectively (35). A retrospective analysis of 768 esophageal cancer patients diagnosed or treated at Amsterdam University Medical Center from 2014 to 2021 explored the performance of ultrasound and ^18^F-FDG PET-CT in detecting cervical lymph node metastases. The study aimed to evaluate the rationale for removing cervical ultrasound from the diagnostic guidelines for esophageal cancer. Results showed that 15% of malignant cervical lymph nodes were detected solely by ultrasound and were missed by ^18^F-FDG PET-CT, confirming the additional value of cervical ultrasound in detecting cervical lymph node metastases. However, the study also noted that routine use of cervical ultrasound increases the number of biopsies performed on benign lesions (36). Another study indicated that while PET-CT has advantages in providing metabolic information for systemic evaluation, its diagnostic sensitivity for cervical lymph node metastases (LNM) in esophageal squamous cell carcinoma was significantly lower than that of ultrasound (45%), although its specificity remained high at 95.6% (37). This finding suggests that the utility of PET-CT in this specific application is limited. Therefore, combining ultrasound with PET-CT in evaluating metastasis in specific cervical regions can further enhance comprehensive diagnostic performance, providing a more robust basis for clinical decision-making.

### 2.3 Ultrasound-guided lymph node biopsy in esophageal cancer

In the diagnosis and treatment of esophageal cancer, accurately determining the nature of metastatic cervical and supraclavicular lymph nodes is critical for formulating individualized treatment plans. Ultrasound demonstrates high accuracy in diagnosing cervical lymph node involvement and can assist in guiding needle biopsy (17). Ultrasound-guided biopsy provides reliable pathological evidence for distinguishing between benign and malignant lymph nodes, making it an essential clinical assessment tool. This approach includes two methods: fine needle aspiration cytology (FNAC) and core needle biopsy (CNB), each with its own advantages. FNAC is simple to perform, minimally invasive, and suitable for cases requiring cytological evaluation. Studies have shown that FNAC has high diagnostic sensitivity and specificity, making it effective for the preliminary assessment of metastatic and reactive lymph nodes. However, FNAC provides limited sample volume, which may affect the accuracy of pathological diagnosis. In contrast, CNB collects complete tissue cores, offering rich histological information and significantly improving diagnostic accuracy. Literature reports that CNB achieves an accuracy of up to 95.83% in diagnosing malignant lymph nodes, with a sensitivity and specificity of 100% and 93.75%, respectively (38). However, CNB carries a higher risk of complications such as bleeding, necessitating careful consideration based on the specific clinical situation. Using ultrasound guidance allows precise localization of target lymph nodes for biopsy, enhancing both the accuracy and safety of the procedure. Additionally, the integration of advanced techniques such as ultrasound elastography and contrast-enhanced ultrasound can provide more precise targeting information. These advancements further improve the efficiency of evaluating lymph node metastasis in esophageal cancer, offering critical support for clinical decision-making.

## 3 Optimization of cervical lymph node zoning in esophageal cancer

### 3.1 Historical evolution of cervical lymph node zoning

The cervical lymph node zoning system has evolved over time in response to clinical demands and advancements in medical technology. In 1981, Shah et al. first divided cervical lymph nodes into five regions (39), with the boundaries defined by surface landmarks distinguishable during surgery or clinical examination. In 1991, the American Head and Neck Society (AHNS) and the American Academy of Otolaryngology–Head and Neck Surgery (AAO-HNS) jointly introduced a cervical lymph node zoning and nomenclature system, dividing cervical lymph nodes into six levels (40). In 2002, this system was updated with several revisions. The boundaries between levels II/III and III/VI were redefined. The division between levels II and III was shifted from the carotid artery bifurcation to a horizontal line passing through the inferior border of the hyoid bone. The boundary between levels III and IV was changed to a horizontal line passing through the inferior border of the cricoid cartilage. The sensory branches of the cervical plexus were introduced as a reference for delineating the boundaries between levels II-IV and V. Subdivisions of levels I, II, and V (into A and B sublevels) were redefined (41). In 2008, the system was updated again (42, 43), incorporating recommendations from imaging specialists to make the boundaries between zones more identifiable on imaging (44–47). In the 2002 definition, the stylohyoid muscle was used as the boundary between levels IIA and IB, but this anatomical landmark is only visible during surgery and not easily recognized on imaging. In the 2008 update, this boundary was revised to a vertical plane passing through the posterior margin of the submandibular gland. The boundary between levels III/IV and VI was redefined from the posterior margin of the sternohyoid muscle to the more imaging-friendly midline of the common carotid artery.

### 3.2 ASHNS/AAO-HNS cervical lymph node ultrasound zoning

**Figure 2:**
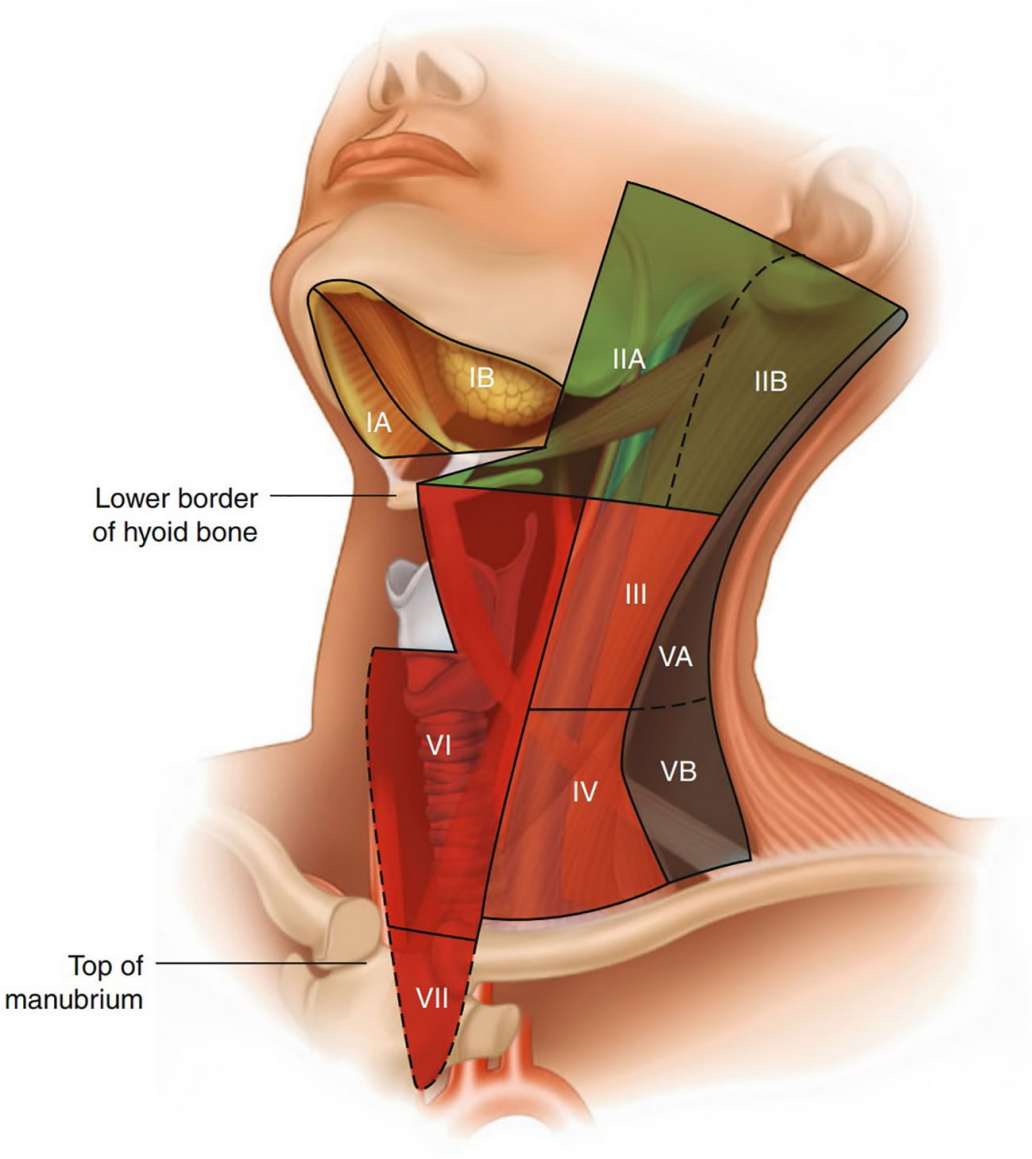
Regional nomenclature of cervical nodes (45).

As illustrated in (**Figure 2**) (45, 48), the ASHNS/AAO-HNS cervical lymph node zoning system divides the neck into seven anatomically defined levels to facilitate standardized evaluation and communication in head and neck imaging. This ultrasound-based classification provides clear boundaries for each lymph node level, which are critical for staging, treatment planning, and surgical decision-making in head and neck cancers, including esophageal cancer with cervical metastasis. Level I (Submental and Submandibular Lymph Nodes): Includes the submental and submandibular lymph nodes, containing approximately 1–14 nodes. These nodes drain lymph from the chin, lips, cheeks, floor of the mouth, anterior tongue, palate, sublingual gland, and submandibular gland. Level I is divided into two sublevels by the digastric muscle: Level IA (medial and inferior) and Level IB (lateral and superior). The boundary between Level I and Level VI is the hyoid bone, while the lateral boundary of Level I is defined by the posterior margin of the submandibular gland.

Level II (Upper Deep Cervical Nodes): This level corresponds to the upper cervical nodes along the internal jugular vein, located below the digastric muscle and extending from the base of the skull to the hyoid bone level. The anterior boundary is the lateral margin of the sternohyoid muscle, and the posterior boundary is the posterior margin of the sternocleidomastoid muscle. This level is often the initial site of metastasis for laryngeal cancer, making it clinically significant. Level II is further divided into two sublevels by the spinal accessory nerve: Level IIA (anteroinferior) and Level IIB (posterosuperior).

Level III (Middle Deep Cervical Nodes): Corresponding to the middle cervical nodes along the internal jugular vein, Level III extends from the hyoid bone to the intersection of the omohyoid muscle and the internal jugular vein. Its anterior and posterior boundaries are the same as those of Level II.

Level IV (Lower Deep Cervical Nodes): These nodes are located in the lower cervical region along the internal jugular vein, extending from the omohyoid muscle to the clavicle. The anterior and posterior boundaries are the same as those of Level II. This level lies within the area formed by the omohyoid muscle, clavicle, and lateral margin of the sternocleidomastoid muscle. Levels II, III, and IV collectively form the internal jugular lymph node chain, which drains lymph from the parotid, submandibular, submental, posterior pharyngeal wall, and anterior cervical lymph nodes, making them key targets in neck dissection surgery.

Level V (Posterior Cervical Triangle Nodes): Includes the lymph nodes of the posterior cervical triangle, also known as the spinal accessory lymph node chain and supraclavicular lymph nodes. The posterior boundary is the anterior margin of the trapezius muscle, the anterior boundary is the posterior margin of the sternocleidomastoid muscle, and the inferior boundary is the clavicle. Level V is further divided into two sublevels by the inferior belly of the omohyoid muscle: Level VA (superior) and Level VB (inferior). Supraclavicular lymph nodes belong to Level VB.

Level VI (Prelaryngeal, Pretracheal, and Paratracheal Nodes): Known as the juxta-visceral nodes or anterior compartment nodes, this level includes lymph nodes around the cricothyroid membrane, trachea (recurrent laryngeal nerve), and thyroid, containing approximately 6–16 nodes. Some also include retropharyngeal lymph nodes in this level. The lateral boundaries are the common carotid artery and the internal jugular vein, the superior boundary is the hyoid bone, and the inferior boundary is the suprasternal notch. Prelaryngeal lymph nodes, located around the cricothyroid membrane, drain lymph from the subglottic region and are clinically significant.

Level VII (Superior Mediastinal Nodes): The AJCC added Level VII in its TNM staging system, corresponding to the superior mediastinal lymph nodes. The lateral boundaries are the common carotid arteries, the superior boundary is the suprasternal notch, and the inferior boundary is the level of the aortic arch.

### 3.3 The necessity of optimizing ultrasound zoning for cervical lymph nodes in esophageal cancer

Current ultrasound zoning standards primarily adopt the cervical lymph node zoning system established by the ASHNS/AAO-HNS. These methods are designed mainly for assessing head and neck diseases. However, esophageal cancer has unique lymphatic drainage pathways and metastatic characteristics, such as those involving supraclavicular and cervical paraesophageal lymph nodes (No. 101 and No. 104 in the JES system). These regions are critically important for staging and treatment decisions.

According to the 12th edition of the Japanese Classification of Esophageal Cancer (JES standard), the definitions of No. 101 and No. 104 lymph node regions are essential for the diagnosis and treatment of esophageal cancer. However, the seven-zone system commonly used in ultrasound reports does not directly correspond to these JES-defined regions. Surgeons specializing in esophageal cancer must manually convert ultrasound findings from the ASHNS/AAO-HN zoning system into the JES system. This process is time-consuming, error-prone, and can compromise the precision of treatment plans.

The Efficacy Index (EI) is a method to quantify the contribution of lymph node dissection to patient survival. It is calculated based on the frequency of lymph node metastasis and the 5-year survival rate of patients with metastases to specific regions. Studies have shown significant differences in EI across lymph node regions for esophageal cancer patients depending on tumor location. For example, in patients with upper esophageal cancer, No. 101R has the highest EI, indicating that dissection of this region is critical for improving survival rates (49). This regional variability in EI underscores the importance of cervical and supraclavicular lymph node dissection in determining effective treatment strategies for esophageal cancer. However, the current ultrasound zoning methods lack systematic optimization for evaluating No. 101 and No. 104 regions, directly affecting the precision of cervical lymph node zoning and subsequently impacting treatment decisions. Research has further indicated that, for cervical esophageal squamous cell carcinoma patients, the metastatic patterns and prognosis of different lymph node regions (e.g., No. 101 and No. 104) vary significantly. Leveraging the advantages of ultrasound technology to optimize the diagnostic criteria for No. 101 and No. 104 regions can enable more precise staging and provide a stronger foundation for individualized treatment strategies. Additionally, advancements in three-field lymph node dissection techniques further support the thorough clearance of No. 101 and No. 104 regions (6). Therefore, optimizing current ultrasound zoning methods to directly correspond to JES-defined zones (particularly No. 101 and No. 104) is essential to improving diagnostic accuracy and efficiency, minimizing discrepancies in treatment decisions caused by zoning differences. This optimization will also provide robust support for personalized treatment approaches, further enhancing patient survival rates and treatment outcomes.

## 4 Standardizing ultrasound-based cervical lymph node zoning with the JES cervical staging system

### 4.1 Naming, numbering, scope, and boundaries of cervical and mediastinal lymph nodes in esophageal cancer

**Figure 3:**
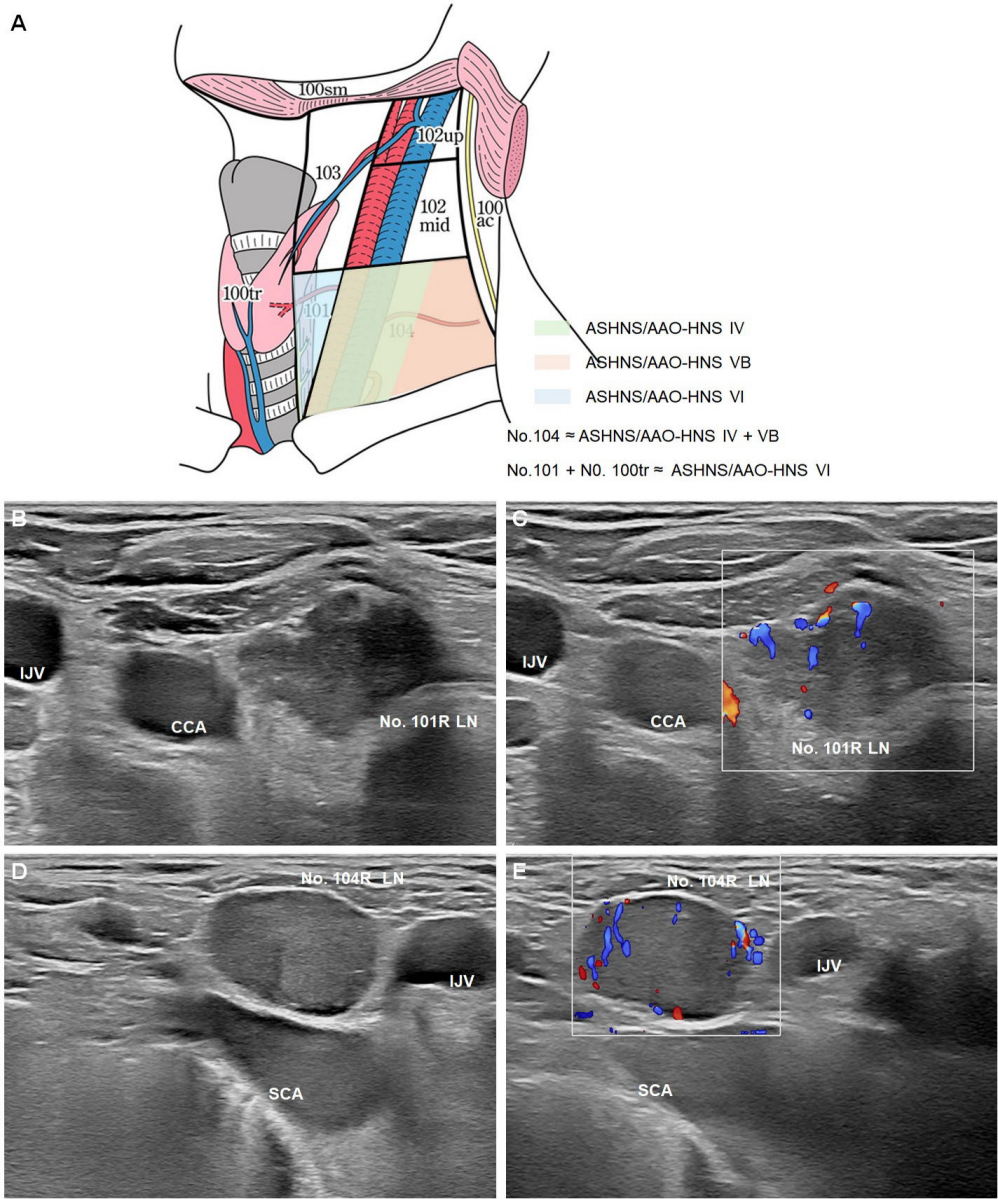
Optimization strategies for ultrasound zoning of cervical lymph nodes in esophageal cancer. **(A)** Schematic illustration of the cervical lymph node partitions relevant to esophageal cancer, comparing the JES classification with ASHNS/AAO-HNS standards (22). The No. 101 and No. 104 lymph nodes are highlighted, with No. 104 corresponding to ASHNS/AAO-HNS levels IV and VB, and No. 101 combined with No. 100tr approximating ASHNS/AAO-HNS level VI. B-mode **(B)** and Color Doppler Flow Imaging (CDFI) **(C)** of the No. 101R lymph node. B-mode **(D)** and CDFI **(E)** of the No. 104R lymph node. LN: lymph node; CCA: adjacent to the common carotid artery; IJV: internal jugular vein; SCA: subclavian artery (SCA).

As illustrated in (**Figure 3A**) (22), the JES classification system provides detailed anatomical definitions for cervical and upper mediastinal lymph node stations relevant to esophageal cancer. These include No. 100–104 stations, each with clearly defined boundaries, locations, and clinical implications. A systematic understanding of these lymph node groups is essential for accurate staging, ultrasound evaluation, and surgical planning in esophageal cancer. No. 100: Superficial cervical lymph nodes, including all superficial nodes in the cervical region. No. 100spf: Superficial cervical lymph nodes located beneath the superficial cervical fascia, distributed along the external and anterior jugular veins. No. 100sm: Submandibular lymph nodes surrounding the submandibular and parotid glands, located anterior to the mylohyoid muscle. No. 100tr: Pretracheal lymph nodes situated within the pretracheal adipose tissue, extending from the hyoid bone to the left brachiocephalic vein, including prethyroid and prelaryngeal lymph nodes. No. 100ac: Spinal accessory lymph nodes distributed along the accessory nerve, located anterior to the trapezius muscle.

No. 101: Cervical paraesophageal lymph nodes, located around the cervical esophagus, including recurrent laryngeal nerve and cervical paratracheal lymph nodes. The lateral boundary is defined by the medial edge of the carotid sheath, with separate zones for the left and right sides.
No. 102: Deep cervical lymph nodes distributed around the internal jugular vein and common carotid artery. No. 102up: Upper deep cervical lymph nodes located between the inferior border of the digastric muscle and above the bifurcation of the carotid artery. No. 102mid: Middle deep cervical lymph nodes located between the carotid bifurcation and the inferior border of the cricoid cartilage.
No. 103: Pharyngeal lymph nodes, located medial to the carotid sheath, between the inferior border of the digastric muscle and the cricoid cartilage, including retropharyngeal and parapharyngeal lymph nodes.
No. 104: Supraclavicular lymph nodes, including lower deep cervical lymph nodes in the supraclavicular fossa, extending from the supraclavicular fossa to the inferior border of the cricoid cartilage. The medial boundary is the medial edge of the carotid sheath, with separate zones for the left and right sides.

### 4.2 Comparison of JES and ASHNS/AAO-HNS cervical lymph node zoning

The differences between the JES and ASHNS/AAO-HNS cervical lymph node zoning systems primarily lie in their scope, definitions, numbering systems, and clinical applications. Specific analyses of No. 101 and No. 104, the most commonly involved lymph nodes in esophageal cancer metastasis, include:

A. Does No. 101 correspond to ASHNS/AAO-HNS Level VI?

No. 101 includes cervical paraesophageal lymph nodes but excludes pretracheal lymph nodes (No. 100tr). Therefore, the scope of No. 101 is narrower than that of ASHNS/AAO-HNS Level VI (prelaryngeal, pretracheal, and paratracheal nodes) (**Figures 3B, C**). JES emphasizes the importance of the tracheoesophageal groove in metastasis for esophageal cancer, a key distinction.

B. Does No. 104 correspond to ASHNS/AAO-HNS Levels IV + VB?

No. 104 refers to lymph nodes in the bilateral supraclavicular fossa and significantly overlaps with Levels IV (lower deep cervical nodes) and VB (inferior posterior cervical triangle nodes) in the ASHNS/AAO-HNS system (**Figures 3D, E**). However, since JES and ASHNS/AAO-HNS zoning systems were designed for esophageal cancer and head and neck tumors, respectively, No. 104 aligns more closely with the specific anatomy of the supraclavicular fossa. It is primarily used to diagnose supraclavicular lymph node metastases in esophageal cancer, whereas Levels IV and VB are considered separate regions in the AAO-HNS system.

C. Does No. 106 correspond to AJCC Level VII?

The No. 106 lymph nodes (e.g., 106pre, 106tbL, etc.) are further subdivided by their specific anatomical locations, such as pretracheal, tracheobronchial angle, and left or right recurrent laryngeal nerve regions. The AJCC Level VII (superior mediastinal nodes) overlaps partially with No. 106pre (pretracheal lymph nodes) in the region between the suprasternal notch and the aortic arch. However, there are differences: Level VII primarily focuses on the overall superior mediastinal region with broader boundaries, whereas JES provides finer granularity. Additionally, the lower boundary of AJCC Level VII is the level of the aortic arch, overlapping with the upper boundary of No. 106tbL (left tracheobronchial lymph nodes). This comparison highlights the need for precise correlation and adaptation between systems to facilitate more accurate diagnosis and treatment planning for esophageal cancer.

Optimizing the ultrasound zoning of cervical lymph nodes in esophageal cancer to clearly define the boundaries of No. 101, No. 104, and No. 106 can help reduce misdiagnosis or treatment discrepancies caused by differences in zoning systems. It plays a vital role in the formulation of comprehensive treatment strategy.

### 4.3 Future optimization strategies for ultrasound-based zoning

The classification of supraclavicular lymph nodes (SCLNs) remains one of the most controversial areas in esophageal cancer staging, with significant geographic variation in interpretation and treatment implications. To address this, our strategy integrates JES-defined anatomical landmarks into ultrasound evaluation, particularly targeting station No.104. This approach seeks to enhance consistency across diagnostic and surgical frameworks.

While integrating the JES-defined lymph node stations into ultrasound-based cervical zoning represents an important first step, further optimization strategies are essential to enhance diagnostic accuracy and clinical applicability. One promising direction is the application of contrast-enhanced ultrasound (CEUS), which provides vascular perfusion information and may improve differentiation of benign versus metastatic lymph nodes, especially in borderline cases (50). Additionally, elastography offers complementary information by assessing tissue stiffness, which can help identify malignant nodes with increased rigidity. In addition, artificial intelligence (AI)-driven segmentation and classification models can support real-time, automated identification of anatomical zones and nodal characteristics, thereby increasing standardization and reproducibility across operators and institutions. These techniques can also assist in mapping lymph node involvement to specific JES zones, facilitating precise treatment planning. Collectively, these technological advancements are expected to strengthen the effectiveness, scalability, and clinical utility of ultrasound-based zoning in the management of esophageal cancer.

In future research, we aim to establish a more esophageal cancer–specific ultrasound-based definition of supraclavicular lymph node involvement. This will be achieved by comparing existing ultrasound zoning standards (e.g., AAO-HNS) with the JES-defined cervical lymph node stations through anatomical and clinical mapping. We also plan to correlate ultrasound findings (such as node size, shape, and vascularity) with surgical and pathological outcomes to evaluate diagnostic accuracy. Advanced techniques like contrast-enhanced ultrasound and elastography will serve as complementary tools. These efforts will help determine whether current zoning systems are appropriate or require modification, ultimately contributing to a standardized, globally applicable ultrasound framework for esophageal cancer staging.

## 5 Conclusion

In the context of multidisciplinary treatment needs, ultrasound demonstrates high accuracy and operational convenience in evaluating cervical lymph nodes in esophageal cancer. At the same time, geographic variations in clinical staging systems, particularly the differences between the Japanese Esophageal Society (JES) system used in East Asia and the AJCC/UICC system commonly followed in Western countries, have led to inconsistencies in the classification and management of cervical and supraclavicular lymph nodes. These differences expose the limitations of current ultrasound zoning practices, which are primarily based on head and neck oncology frameworks and may not fully align with the lymphatic dissemination patterns of esophageal cancer. Introducing zoning optimization based on the JES system and using the efficacy index as a guide for ultrasound-based diagnosis can not only improve diagnostic accuracy but also provide robust data support for personalized treatment strategies. Future research should integrate artificial intelligence algorithms and real-time imaging technologies to advance ultrasound zoning toward greater precision and intelligence. This approach could significantly improve the prognosis of esophageal cancer patients and elevate precision medicine and multidisciplinary collaboration to a new level.
